# Preventing Biofilm Formation and Development on Ear, Nose and Throat Medical Devices

**DOI:** 10.3390/biomedicines9081025

**Published:** 2021-08-16

**Authors:** Dan Cristian Gheorghe, Andrei Ilie, Adelina-Gabriela Niculescu, Alexandru Mihai Grumezescu

**Affiliations:** 1 “Carol Davila” University of Medicine and Pharmacy, 050474 Bucharest, Romania; gheorghe.dancristian@gmail.com; 2 “M.S. Curie” Clinical Emergency Hospital for Children, 077120 Bucharest, Romania; 3 Faculty of Engineering in Foreign Languages, University Politehnica of Bucharest, 060042 Bucharest, Romania; andrei.ilie.97@gmail.com (A.I.); niculescu.adelina19@gmail.com (A.-G.N.); 4 Faculty of Applied Chemistry and Materials Science, University Politehnica of Bucharest, 060042 Bucharest, Romania; 5 Research Institute of the University of Bucharest—ICUB, University of Bucharest, 050657 Bucharest, Romania; 6 Academy of Romanian Scientists, 3 Ilfov Street, 50044 Bucharest, Romania

**Keywords:** otorhinolaryngology, tissue engineering, voice prosthesis, cochlear implants, tracheal stents, MSC, biofilm

## Abstract

Otorhinolaryngology is a vast domain that requires the aid of many resources for optimal performance. The medical devices utilized in this branch share common problems, such as the formation of biofilms. These structured communities of microbes encased in a 3D matrix can develop antimicrobial resistance (AMR), thus making it a problem with challenging solutions. Therefore, it is of concern the introduction in the medical practice involving biomaterials for ear, nose and throat (ENT) devices, such as implants for the trachea (stents), ear (cochlear implants), and voice recovery (voice prosthetics). The surface of these materials must be biocompatible and limit the development of biofilm while still promoting regeneration. In this respect, several surface modification techniques and functionalization procedures can be utilized to facilitate the success of the implants and ensure a long time of use. On this note, this review provides information on the intricate underlying mechanisms of biofilm formation, the large specter of implants and prosthetics that are susceptible to microbial colonization and subsequently related infections. Specifically, the discussion is particularized on biofilm development on ENT devices, ways to reduce it, and recent approaches that have emerged in this field.

## 1. Introduction

The field of otorhinolaryngology is developing fast due to the wide variety of damages and their unexplored solutions. Completing many fields with complex components is a big advantage, bringing multiple materials, tests, and high interest to this medical specialization. Other fields, such as 3D printing and telemedicine, have had an impact by multiplying the options and offering new perspectives for this healthcare branch. The combined existing information has helped the field develop quickly and offers new techniques that can be utilized independently or allow the expansion of existing options. However, biofilm formation remains a common issue in this application area, as it can promote the development of antimicrobial resistance and generate weakness points for any device or action [1,2]. Specifically, this phenomenon is encountered in patients who undergo implantations of exogenous materials; thus, they are susceptible to postoperative infection risks that often result in prolonged hospitalization, development of various diseases, and increased morbidity [3].

Biofilms can be defined as microbial communities rooted in a 3D extracellular matrix (ECM), generating a well-organized structure that offers self-protection and enhanced survival abilities but is responsible for several human infections [4,5]. The first domain in which the term “biofilm” was utilized was marine microbiology to differentiate planktonic and adherent microbial cells, later becoming the main concern for the environment and human health. The main role of the ECM in the biofilm is to enclose the bacterial colony and aid the adhesion to the substrate acting as a scaffold and defending the cells against environmental threats. The necessary energy for the pathogens are sugars which influence the ECM and the acidity of the microenvironment, thus aiding in changes of the medium and combined multispecies efforts [6,7].

In the clinic, the self-protecting behavior of biofilm typically leads to chronic, nosocomial, and medical device-related infections [8]. Therefore, the prevention of biofilm formation or the reduction of existing biofilms in clinical settings represents a critical aspect of effective care, and new strategies have to be considered. Only through a deep understanding of the mechanisms of formation of biofilms and recognizing their major role in a significant portion of human diseases is it possible to envisage novel potential approaches to treat and prevent biofilm development [9]. 

Thus, this review comprehensively describes the factors contributing to biofilm formation, the infections triggered by biofilm development, antimicrobial agents and antimicrobial resistance of pathogenic communities. Moreover, the enhancement of biomaterials through surface modification techniques is presented, further particularizing the discussion for ENT devices, such as voice prosthetics, cochlear implants, and tracheal stents.

## 2. The ECM Role in Biofilm

The ECM augments the water retention, inorganic ions and organic compounds are adsorbed, surplus carbon is stored, and horizontal gene transfer occurs. It additionally influences the polymicrobial interface, mechanical equilibrium, resistance to antibiotics, and biofilm architecture development [6,10,11]. The biofilm ECM is composed out of macromolecules like extracellular DNA (eDNA), proteins, and polysaccharides [10]. The general model was built for aquatic systems and the medical field. Analysis of natural biofilms, artificially obtained ones and computer models all indicate that the biofilm composition is mainly defined by the predominant substrate concentration and hydrodynamic circumstances. The structure can also be influenced by protozoa feeding on natural and industrial environments [11,12].

The ECM produces proteinaceous elements for the biofilm pattern, such as amyloids, which can be defined as structured protein clusters primarily known to be correlated completely with the biochemical symptoms of human diseases [13], but a distinction between the functional and pathological amyloids emerged with the discovery of their functionality, though they share biochemical and biophysical characteristics [14,15]. An abundance of functional amyloids is crucial in biofilm expansion, the evolution of aerial structures, alteration of melanin synthesis, genetic data transmission, scaffolding, and epigenetic control of polyamines [15,16].

## 3. Amyloid Influence in Biofilm ECM

Production of functionalized amyloids is realized using specific pathways. The variety of functional amyloids through cellular life indicates that the amyloid fold is an indispensable protein folding state. There have been several studies about the mechanisms of regulation for many functional amyloid and amyloid-like structures, incorporating findings concerning spatially controlled nucleation mechanisms, biological inhibition of amyloids, aggregation reversibility, and protease cleavages that can rapidly generate functional amyloids, determining the exact cellular timing for the amyloid formation [17,18,19]. In addition, there are secondary processes like fragmentation and nucleation occurring on the fibril area, which are less noticeable for functional amyloids than pathological amyloids. This indicates the purpose of the folded monomer for the types directing fibrillation [20]. Among other recent updates, in the catalog for amyloid structures, the cross-α structure of phenol-soluble modulin, a functional amyloid generated by *S. aureus*, has been determined [21].

There are many prospects of improvement when we discuss the depth of this branch, one of which is the capacity of bacterial amyloids to tap into cross-section human amyloid reserves, another being the chance that bacterial amyloid-induced inflammation will engage human amyloidosis. This will generate insights on the impact of the exposure for an extended period to bacterial amyloid. Another aspect of interest is the bacterial amyloids’ inertness level and their by-products because of their potential applications in the biomedical field [22]. 

The contribution of amyloids to the biofilm matrix is focused towards the adhesion on abiotic and biotic surfaces, growth of hydrophobicity, and colonization stimulation. Additionally, they increase the stability of the structure, offer environmental stress resistance and protect the matrix from phage particles and matrix-damaging enzymes [23]. Besides the functionality, there are links between bacterial amyloids and pathological diseases, augmenting stomach inflammation, causing host cytolysis, affecting neuronal inflammation and the clustering of cerebral amyloids [24]. There is a term for inducing a host immune response, which is microbial-associated molecular patterns (MAMPs) [25]. The reason behind studying amyloids, and the regulatory system, is the crucial part they play in the development of biofilms and disease. 

A report from the National Institutes of Health, USA, shows that 65% of acute microbial infections and 80% of chronic microbial infections are related to biofilm development [26]. Biofilms with streptococcal provenance that trigger chronic tonsils infections could intensify psoriatic symptoms. Studies have concluded that patients suffering from moderate to acute, chronic plaque psoriasis reacted well to long-term treatments with penicillin, ameliorating skin lesions [27]. Other reports focused on patients who undertake transvaginal mesh surgeries or hernia operations, given that 20 million inguinal hernia repairs are reported yearly in the world, some of which formed autoimmune diseases, systemic lupus erythematosus (SLE) included. There is a hypothesis that biofilms correlated with mesh break tolerance can cause an autoimmune response. Furthermore, multiple biofilm species have been detected that adhered to the implanted mesh [28,29,30].

Several oral tissues, including gingiva, can present pathologic trends evidenced as symptoms or expressions of systemic disorders, requiring additional diagnostic and interdisciplinary care [31]. The epidemiological information presents a low occurrence in regional diseases like oral lichen planus (oLp) (1–2%), Sjögren’s syndrome (SjS) (0.5–1%), and SLE (0.05%), and even for some scarce occurrence such as epidermolysis bullosa (EB) (up to 0.001%), systemic sclerosis (sSc) (up to 0.03%), and pemphigus (Pe) (0.05%), patients suffering from these variations require extensive dental treatment and prosthesis [32,33,34]. 

Among the bacterial functional amyloids, there is Curli, whose expression and translocation are determined by Curli-specific genes (Csg), which are coded by CsgBAC operon [35,36]. Between some of the roles of Curli in some pathogenic bacteria, we can include colonization, invasion of the cells, and intrinsic response activation. The CsgBAC operon uses CsgB minor Curli subunit encoding, CsgA for major Curli, and periplasmic protein CsgC. When exposed to good biofilm generation conditions, the Curli fabrication is started by CsgB, providing the template for CsgA amyloid generation on the exterior surface. The role of CsgC is to maintain the CsgA in soluble conditions inside the cells. An additional operon is CsgDEFG, which encodes the augmenting proteins for curli production: CsgD is a master regulator protein from the transcriptional family that controls Csg expression, the modulation occurs at the transcriptional level as well as post-transcriptionally using sRNAs and regulatory proteins; CsgG is a nonameric lipoprotein found in the exterior membrane which contributes to the generation of CsgA and CsgB; CsgE is a protein that modifies CsgG to add specificity to the depending sections, and it also helps CsgA to stay in a soluble condition while found in cells; CsgF is an adaptor which augments the amyloid generation to the surface of the cell [37,38,39].

Proteinaceous membrane-less organelles (PMLO) are obtained by liquid to liquid transitions that are undergone by intrinsically disordered proteins (IDPs) and intrinsically disordered regions (IDRs) reacting inside the nucleus or cytoplasm compartments because of the lack of complexity and great net charge. The main motives for PMLOs formation are the medium changes and frequency of liquid-liquid phase transition led by the main sequence of the protein components [40,41]. One reason that allows the PMLOs to engage in a wide specter of molecular interactions is their dynamic nature. The existence of disordered proteins related to PMLOs in various organisms indicates IDPs/IDRs as a conserved unfolding factor [42]. Also, the IDPs take part in the transition and generation of membrane-less organelles in plants [43]. Another aspect regarding the IDPs is that the transition can trigger the development of structured homo-aggregates or amyloids linked with neurodegenerative illnesses [44].

## 4. Biofilm-Related Infections

**Figure 1:**
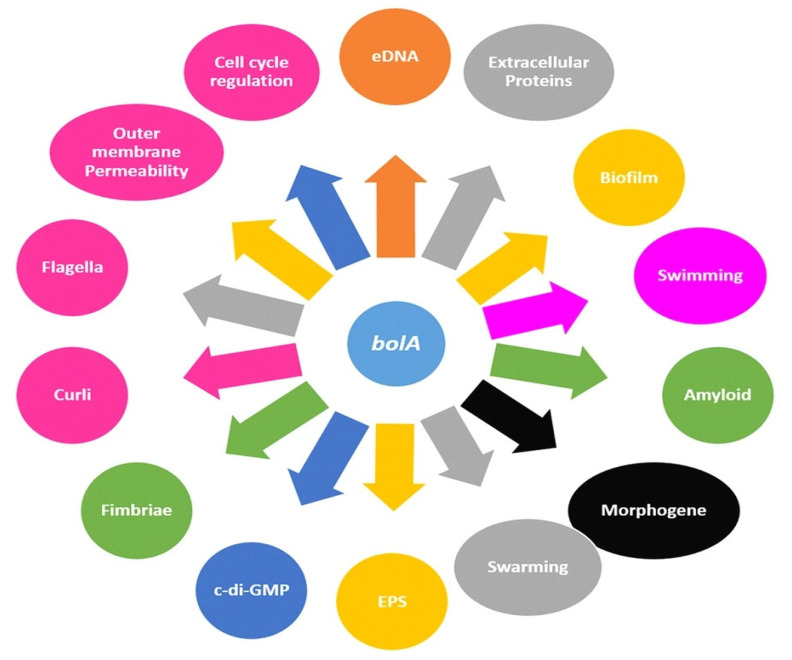
Cellular mechanisms in *E. coli* connected to the *bolA* gene/BolA protein (Reprinted from an open-access source) [45].

Contributing to the wide range of human infections, such as meningitis, pyelonephritis, septicemia, and cystitis is *Escherichia coli* (*E. coli*), which is also a factor in urinary tract infections triggered by uropathogenic *E. coli* (UPEC) [45,46]. The strains of UPEC generate biofilms, precisely intracellular bacterial communities (IBCs), between the superficial cells or on the epithelial surface of the bladder and catheters, while extraintestinal infections have a high percentage of mortality within a month from infection [47,48]. As a result, the biofilm is covered by a matrix made from an exopolymeric substance, which acts as a diffusive barrier preventing the infiltration of high molecular mass antimicrobial components and maintains a nutrient gradient that controls the growth rate that is also influenced by *bolA* [49,50]. More specifically, BolA protein has been associated with pleiotropic effects over cell physiology, controlling a variety of phenotypes, including bacterial morphology, fimbria-like adhesins, Curli fiber formation, flagella formation, and membrane permeability [45,51,52,53] (Figure 1). 

As a DNA-binding regulator, *bolA* overexpression in *E. coli* promotes a spherical morphology which causes a reduction in the surface exposed to unfavorable medium conditions. Thus, *bolA* helps in cell adaptation and survival under adverse growth conditions, contributing to the formation of bacterial biofilms [45,50,51,52,53,54]. 

**Figure 2:**
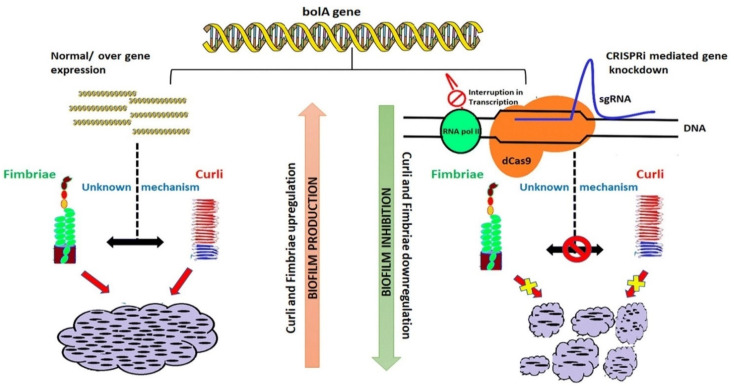
Model of the influence of *bolA* on the Curli proteins and biofilm (reprinted from an open-access source [45]).

The biofilm formation process for *E. coli* can be influenced by multiple variables, including Curli and self-transporting proteins such as cellulose and polysaccharides. One of them is phosphoethanolamine cellulose, and its presence could diminish Curli stimulated immune response [55]. Furthermore, Curli fibers can be programmed to support a wide selection of functional biomolecules that can be used to supply self-sustaining therapeutics or obtain the environment’s bioremediation [56]. Figure 2 presents an established model that displays the *bolA* gene mechanism’s operation that influences the Curli cells and modulates the biofilm formation [45].

## 5. Antimicrobial Agents and Antimicrobial Resistance

**Figure 3:**
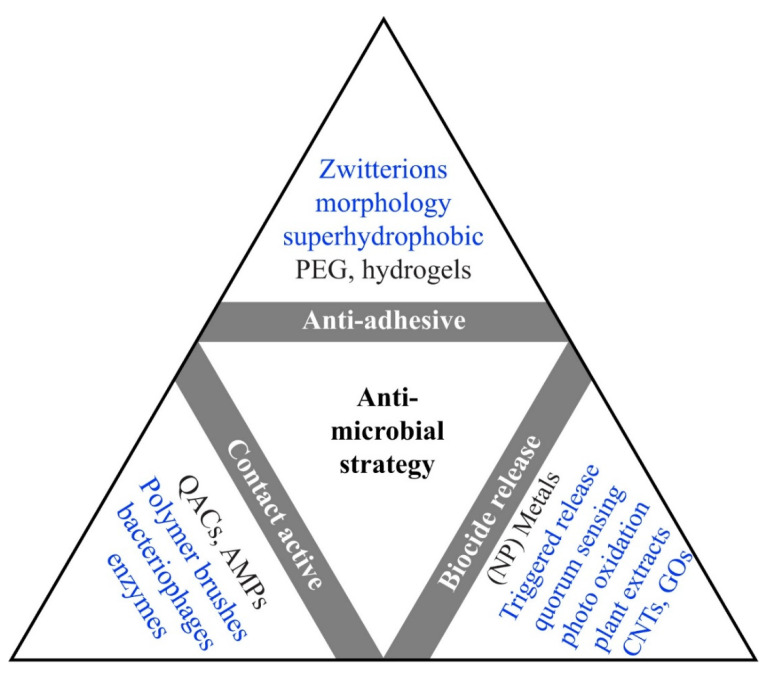
Illustration of in use (black) and hypothetical approaches(blue) of antimicrobial coatings categorized by their operating theory. Abbreviations used in the figure are quaternary ammonium compounds (QACs), Carbon nanotubes (CNTs), polyethylene glycol (PEG), antimicrobial peptides (AMPs), and graphene oxides (GOs) (reprinted from an open-access source [67]).

Various types of antibiotic substances are available now with bactericidal or bacteriostatic effects, but the availability and massive use enhanced the discerning pressure on bacteria [57,58]. Therefore, antimicrobial resistance (AMR) had a growth in time for pathogenic bacteria resulting in a potentially fatal outcome [59,60]. There is a need to discuss the AMR topic with the general public and the medical field to obtain a balanced consumption of antibiotics and thus reduce the incidence of resistance [61,62]. Developed countries end up having larger stocks of antibiotics available due to good administration [63]. For example, some 800 tons of antibiotic treatments are used by Germany’s population per year, from which 600 tons are utilized for ambulatory care, and more than half are prescriptions [64]. But Germany is a country with low consumption of antibiotics compared to the reserve used, which is high. There have been international agreements proposed to guarantee the availability of antibiotics for the next generations [65]. There have been reports of increasing resistance to tetracycline, erythromycin, and ciprofloxacin. For example, a non-healing wound provides an ideal environment for gene transfer, aiding antibiotic-resistant pathogens [66]. In addition, the bacteria in the biofilm can influence bacterial development on and in medical devices and is also a facilitator for periodontitis and endocarditis [67]. The studies concluded that it is vital to obtain alternative methods to traditional medication to contain the biofilms’ drug resistance, promoting the survival of bacteria. Differences have been noted relating the cell walls of Gram-positive and Gram-negative bacteria regarding the composition and the layer of murein, which can be a reason for the growth of minimal inhibitory concentration (MIC) values between *S. aureus* in comparison with *E. coli* [68]. The Gram-positive bacteria can neutralize the cell wall partially to withstand antimicrobial peptides (AMPs). Several ways of exploiting the AMPs properties, such as Magainin H2, have a high level of hydrophobicity and present a good permeabilization action on lipid bilayers. Thus, experimental and theoretical studies investigated how AMPs and lipid bilayers interact, concluding that, at first, the lateral growth of the membrane caused by interactions with the AMP is inhibited, but in the end, it generates new bilayer regions that have defects in their membrane [69,70]. Another feasible option is the combined use of AMPs and conventional antibiotics, showing synergy and lowering microbial resistance [71]. Particularly, these work in tandem as AMPs form pores in the bacterial membranes or inhibit bacterial macromolecular functions, while antimicrobial agents aid in keeping the pores open for longer periods, prevent pore repair, disturb intracellular functions at greater levels, or act upon independent bacterial killing mechanisms [72]. Moreover, there have been reports showing the anticarcinogenic aspect of the AMPs and the capacity to destroy unhealthy cells while exhibiting minimal toxicity [73,74]. Because it is difficult for the bacteria to develop resistance to AMPs, they found a more extensive use than antibiotics in the food and farming industry [75]. In Figure 3, the operational idea also guides after the application of the technique. For example, QACs activity from a chemical point of view occurs in solution as well as to a surface [67]. 

## 6. Surface Modification

**Figure 4:**
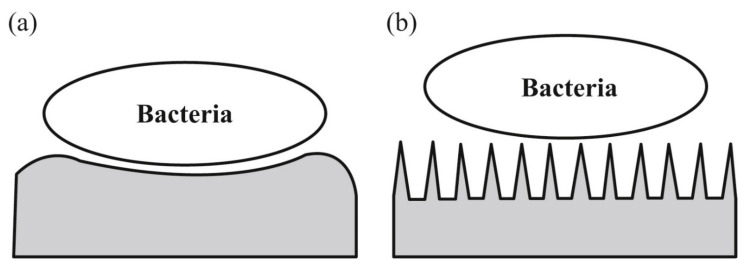
(**a**) Micron-sized elements can favor bacterial adhesion, (**b**) nano-sized features can produce problematic topographic adhesion conditions (reprinted from an open-access source [67]).

A promising strategy for preventing biofilm formation is the surface modification of implants and medical devices [76]. Of particular interest is the topographical adjustment to create anti-adherent surfaces. The topography influence is displayed in Figure 4. In Figure 4b, the surface is constituted from surface structures smaller than the cells, thus inhibiting the adhesion and enhancing the capacity to clean the surface [67].

Many nanofabrication techniques can be utilized for generating anti-adherent surfaces. A general classification divides them into physical fabrication techniques and chemical techniques. The physical techniques available today are machining (milling, polishing, or turning), sandblasting, laser treatment (3D structures can be obtained), sputtering deposition, plasma spraying, or ion-beam assisted deposition. The physical modifications realize a dry conversion of passive implants to smart surfaces, augmenting tissue regeneration [77]. The chemical modifications that can be currently employed are acid etching (solvent cleaning, chemical etching, or passivation treatments), alkali treatment, anodic oxidization (electrochemical deposition realized in the electrolyte), micro-arc oxidation, wet chemical deposition (“sol-gel”), chemical vapor deposition and self-assembled monolayers. 

Another increasingly researched alternative for preventing bacterial cell attachment is the use of antimicrobial coatings. The coating materials can have intrinsic antibiofilm properties or embed and elute antimicrobial drugs [8,76,78]. However, one disadvantage of biocide-releasing surfaces can be the risk of toxicity and inconsistency in the activity. On this note, the actual preference in terms of options is catalytically active surfaces that can rejuvenate reactive oxygen species with the aid of UV light. Alternative approaches are the triggered release set to a threshold or surface coatings such as carbon nanotubes, graphene, or diamond-like carbons. AMPs can be utilized by polymer brushing fixation to the surface and macrophage anchoring to the implant, which is cost-effective but was neglected in the past due to their provenience [67]. Even though many techniques can be utilized, the best way to obtain an optimal surface is by combining the beneficial features of several techniques and materials [79].

## 7. Voice Prosthetics Rehabilitation

In 2018, 177,422 persons were diagnosed with laryngeal cancer, causing 94,771 deaths worldwide [80]. The usual incidence ratio between male and female patients for laryngeal cancer is 5:1, but some parameters can create an imbalance, such as the stage of diagnosis, which is usually higher for male patients, representing a difficulty for organ preservation [81]. There have been attempts to protect the larynx in hypopharyngeal carcinomas with organ preservation protocols, but impediments such as the loco-regional recurrences in partial laryngopharyngectomy (TLPP) or total TLTP that are related to worse results in comparison to primary laryngopharyngectomy [82,83]. Salvage protocols encounter another problem besides the recurrence, namely the high rate of complications, such as pharyngocutaneous fistula (PCF), dysphagia, and pharyngeal stenosis, experienced by 28.9%, 18.6%, and 14.2% of the patients, respectively. These health-threatening complications can be revised with surgery [84,85,86]. Aside from organ preservation, fundamental limits concern the patients, such as severe dysphagia and potential aspiration leading to pneumonia. Hence, there is a need to evaluate the functionality of voice and swallowing capacities to select the optimal treatment [87,88]. 

Nonetheless, the standard treatment for this type of cancer was total laryngectomy (TL) combined with radiotherapy (RT) for patients who have advanced laryngeal cancer, but therapies such as chemotherapy followed by RT were tested for the preservation of the larynx, thus being the first step in organ-preservation protocols [89,90]. A consequence of TL is voice loss which requires vocal rehabilitation methods, one of which is tracheoesophageal (TE) puncture with the insertion of a voice prosthesis (VP) [91]. 

Thus, the main objective after laryngectomy is speech rehabilitation [92]. The rehabilitation rate is influenced by the type of intervention utilized. Some studies show percentages for patient voice recovery in the range of 80% for patients who have undergone laryngectomies and under 50% for pharyngectomy, which requires a free flap rebuilding [93]. Multiple studies are focusing on the endpoints for laryngectomy or laryngopharyngectomy, approaching variations of flap types [94]. The speech outcomes in literature were one year after surgery for efficient tracheoesophageal speech, and a time interval consistency can be observed between particles [95]. There is difficulty in obtaining any major discrepancies between primary and secondary TE punctures because, in the past, the secondary was the one preferred in voice recovery [96]. Salvage total laryngectomy shows growth in speech recovery consistently [97], but there are different results for some particularities, like the influence of the reconstructional typology impact [98]. Breakthroughs have been made regarding the link between the dilatation necessity and its association with the gastrostomy tube and the postoperative fistula formation influence by the dilatation [99].

**Figure 5:**
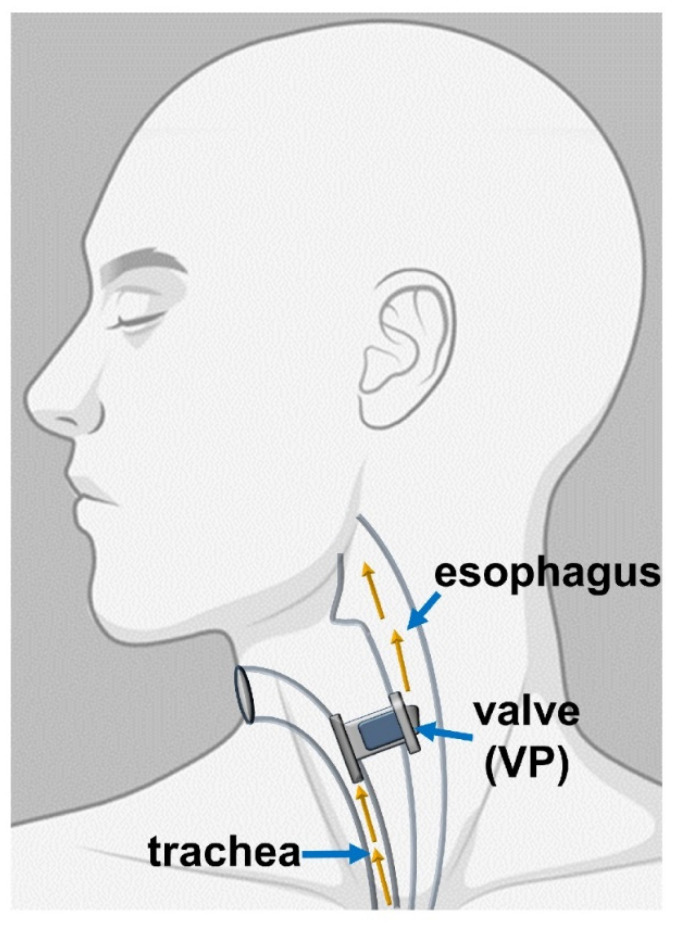
Representation of the placement of the VP (Reprinted from an open-access source) [83].

Some VPs are commonly used because of their reliability, such as Provox and Bloom-Singer, having comparable lifespan and quality. Even though fungal biofilms can lead to infections, the main concern to VPs is the local inflammation of the tracheoesophageal fistula [100]. The TE fistula is utilized to re-establish the link between the superior gastrointestinal tract and the trachea, which TL has disrupted. Figure 5 displays the working principles of the VP, which has the role of a one-way valve permitting the gas flow from the trachea to the esophagus and protecting the trachea from fluids and esophageal matter [83].

The main disadvantage of VPs composed of silicone polymers is the fungi and biofilm colonization [101]. Analysis of biofilm formations on failed devices shows yeast strains such as *Candida glabrata*, *Candida tropicalis*, *Candida albicans*, and *Candida krusei*. The adherence can be controlled by host immunology, surface topography, and genetic profiling [102]. Fungi such as *Candida albicans* act as an opportunistic pathogen, producing superficial infections and can develop systemic infections for immunocompromised patients [103].

Several improvements can be made to advance further with the techniques, such as the resistance of the polymeric material to perform better and sustain deformation and have a lower possibility of cracking. Another good way to develop is by enhancing the antimicrobial properties, which can be obtained by adding nanosystems and chemical compounds to decrease biofilm formation [104,105]. 

Several approaches can be considered in nanosystems’ direction, but the main is using magnetic nanoparticles (MNPs), which can influence microbial cell sensitivity, limiting biofilm formation. In addition, MNPs enhance the fungicidal effect, facilitating peptide transfer into cells and dislocating the fungal membrane. This technique responds well when combined with AMPs or antibiotics, resulting in different results for the chosen method. AMPs can reduce drug resistance, but it is an expensive alternative, in which case the antibiotics can be utilized. Antibiotics can be utilized in lower doses, thus slowing the rate of antimicrobial resistance [104,105].

## 8. Cochlear Implants

Some of the most utilized implants besides nose and throat implants are those for the ear [106]. This category can include cochlear implants (CI) and bone-anchored hearing aids (BAHA), middle ear transducers, tympanostomy tubes, and auditory brainstem implants. The discovery of CI developed the management of auditory problems around the world [107]. At its core, the operational principle has a platinum-based electrode, which stimulates the cochlear nerve and the spiral ganglion cells turning acoustic sounds into an electric signal. The position can influence the success due to the safety of the receiver-stimulator package, thus reducing trauma [108]. 

Some options surpass the use of solely CI, such as bimodal hearing. Studies show a different fitment of the hearing aid (HA) which is independent of the CI and show benefits such as better sound localization, speech intelligibility, sound quality, and lower listening effort [109,110]. 

Biofilm growth on the CI can generate chronic and refractory infections. One of the used methods of inhibiting the formation and reduce the preexisting biofilm is bioactive glass. The usual bacteria found on the electrode and the polymeric surfaces are *P. aeruginosa* and *S. pyogenes*, while *S. epidermidis* and *S. aureus* have a lower presence in the biofilm for this kind of implant. Studies found patterns of biofilm formations for species and materials [111]. There are great examples of bioglass such as S53P4, which contains sodium oxide, phosphorus pentoxide, silicon dioxide, and calcium oxide and has been tested successfully for osteoinduction and reduction of bacteria [112,113].

There has been a technological flux established for solutions such as mats made by piperacillin-tazobactam (PT) elution from electrospun poly(e-caprolactone) (PCL)-polyethylene oxide (PEO)-PT PCL. Evaluation of the obtained materials efficacity was realized by SEM using the dead/live staining technique [114]. Other approaches such as mesenchymal stem cells (MSCs) utilization that coat the electrode enhancing drug delivery are interesting. Composite coatings combined with alginate reduce the electrode’s insertion forces and protect spiral ganglion neurons [115,116]. The purpose of the alginate in the coat is to ensure biocompatibility and limit cell migration [117].

Another option for limiting biofilm formation is the use of a hydrophobic coating that also helps to reduce the probability of corrosion. Such coatings can be obtained by the use of zinc oxide (ZnO) nanowires. Although different substrates influence the contact angle for different substrate materials, ethylenediaminetetraacetic acid (EDTA) is an anticoagulant applied before and after the ZnO nanowire coating. Besides, there have been reports of suitability for ZnO displaying the inhibition of bacterial development in normal conditions and using UV light [118].

## 9. Tracheal Stents

The trachea is a flexible tube with cartilages that links the superior respiratory system to the lungs, acting as a ventilation system. Issues appear when damage is done to the trachea and problems occur [119]. Two types of damage can be done to the trachea: tracheal stenosis, which reduces the trachea in diameter, and tracheomalacia, defined as weakness of the tracheal walls [120,121]. Tracheal stenosis can appear due to inborn defects, tumors, injuries, and inflammation. In contrast, tracheomalacia can occur because of surgical wounds and chronic infections such as tracheostomy infection, polychondritis, inhaling toxic vapors, gastroesophageal reflux, and, most frequently, congenital malformation. Left untreated, it leads to tracheal collapse with severe respiratory issues [122,123].

The existing options for this prosthetic have some setbacks like tissue integration and inflammatory response. The mimetism of the properties is crucial to obtain a good quality implant [124]. One approach is to equip the implant with the optimal growth factors, scaffolds, and cells for the best results in tissue regeneration [125,126]. When flexibility modulation is a priority for cartilaginous tissues, chondrocytes derived from MSCs are a great option [127]. Another important aspect is the number of cells, specifically co-cultures of tracheal epithelial cells. But, other cells of interest, such as rho-associated kinase and feeder cells, can augment cell division and differentiation rate in vitro and in vivo [128]. Some studies used co-cultured cell lines to obtain fully operational new tissue with enhanced epithelial growth with smooth muscle cells and did not report any tumor development compared to embryonic or fetal sources [129].

There are options such as 3D printing that have started to gain much interest lately and approach the problem differently. It is known that 3D printing can be used to create scaffolds, and a part of that is a good way to create a structure for the co-cultured cells, such as bone marrow isolated MSC, endothelial cells, and fibroblasts. This type of technique performs well in vivo with nutrient flow offering growth in strength. Catheters that contain implants with bioengineered tracheal tissue are promising [130,131].

The alternative is additive manufacturing (AM), which has been applied in many domains, including the biomedical one [132,133]. Some of the advantages are the ease of reproducibility and the capacity to produce complex models using biocompatible items producing devices that can be utilized internally and externally [134]. In the past, 3D-scanning and 3D-imaging were used for diagnosing, but now several models are printed with the help of computer assisted design (CAD) programs to obtain a printable model with the desired dimensions and can be modified to suit the patient [135]. Because the technique received much attention and abundant data, it made possible the production of customizable prosthetics used in surgery [136].

When stents for the trachea are made with AM, the usual approach uses the fused deposition modeling (FDM) method of casting molds with biocompatible silicone. One drawback is the surface finishing which can be adjusted by a chemical treatment realized after the casting [137]. The molds are cast to obtain anatomical stents, augmenting the stability and reducing the stress exerted on the trachea [138]. In addition to AM, an alternative method would be rapid tooling which utilizes similar materials [136].

**Figure 6:**
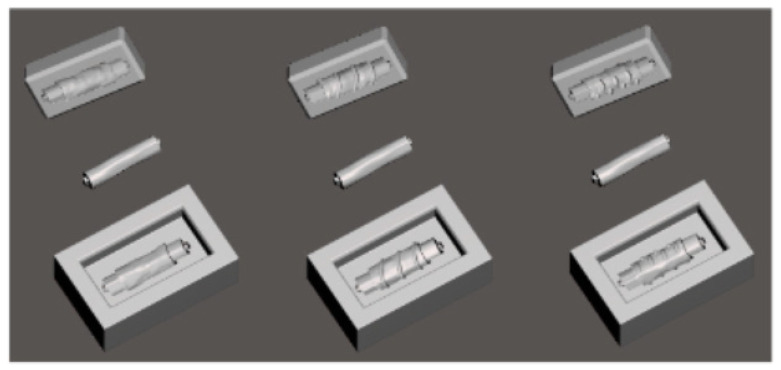
Printing molds and middle pieces (Reprinted from an open-access source) [138].

In Figure 6, we can observe molds for the stents designed to hold a middle piece for the hollow middle to be obtained on the desired dimension. The image also displays the added patterns to match the anatomical specifications to the geometry [138].

One suitable material used for this application is SILBIONE RTV 4439 A&B, a two-component silicone with a curing temperature of 23 °C with the use of a polyaddition reaction [139]. The material comes in two separate components to adjust the ratio for the required application, but in the case of tracheal stents, this particular silicone can be utilized with a ratio of 1:1. The polymerization occurs without dissipating heath, and degassing is done to prevent air insertions. Temperature variations can influence the curing process, and the optimal time is 20 min at 23 °C [140]. Table 1 displays the essential properties of silicone.

**Figure 7:**
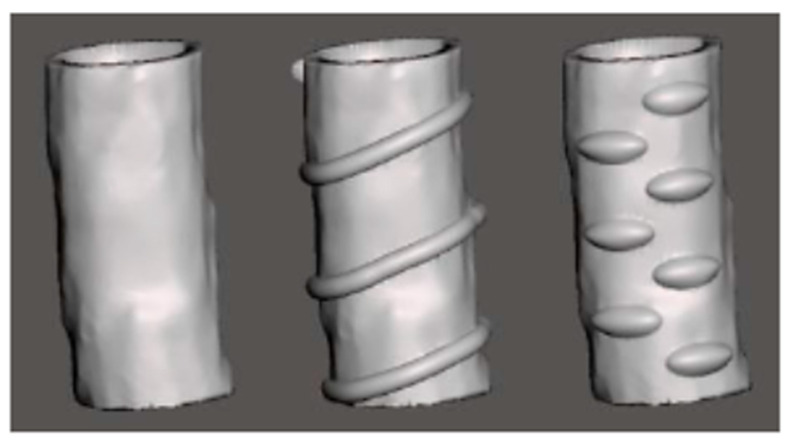
3D models for tracheal stents (Reprinted from an open-access source) [138].

In Figure 7, we can observe the 3D models of the finished products, which propose three geometries that can be utilized in this application [138].

There are multiple approaches in cellular engineered implants that help the growth and integration of the implant but can be susceptible to infections. The methods that can be used in this direction are decellularized donor trachea or biodegradable shells functionalized with autologous stem cells, which repopulate the graft by differentiation and enrollment of circulating cells [126]. Another angle presented is utilizing an acellular source such as xenogenic acellular dermal matrix (ADM), which can be implanted when adenoid cystic tissue resections. This results from tracheal tissue regeneration without infections and avoiding dyspnea [121]. The criteria that are followed for the success of these implants include creating a barrier to prevent the infection. Approaching the patient’s autologous cells is a method that offers alternatives in airway reconstruction, organoid units being of interest to this kind of application [129].

## 10. Conclusions and Future Perspectives

A common denominator of many medical devices is the formation and development of biofilms, and ENT implants and prostheses are no exception. That means there is much room for improvement in this area. Strategies such as designing improved therapeutic formulations, functionalizing materials’ surfaces, or adjusting their topography have shown promising results in reducing microbial colonization and associated infection rates. Other important aspects that have to be considered for creating ideal devices are their ease of insertion, enhanced flexibility, and ability to regenerate tissue while being tailored for the patient.

To tackle these requirements, methods such as 3D printing are more popular and offer a major advantage in adjustability, respecting anatomical shapes with ease. Additive manufacturing is another procedure that has been implemented to generate cost-effective devices and viable solutions. Another direction of biofilm reduction is represented by the study of amyloids, which are heavily involved in biofilm formation and can reduce it with the help of reverse engineering. In addition, chemical surface treatment and functionalization have been in focus lately due to the decrease in research expense and good results for in vitro testing.

Considering every aspect, several techniques are envisaged for this field, generating cost-effective solutions that can impact the domain. The results revolve around already implemented methods, but we can observe breakthroughs in techniques such as plotting specific shapes and utilizing multiple materials to reduce the biofilm and ensure the optimal healing of the tissue. Multiple strategies can be added that are antimicrobially oriented, such as biocide release, anti-adhesive materials and bacteriophage enzymes complementary to the field. Thus, it can be concluded that emerging interdisciplinary research can bring unprecedented results in fighting against biofilm formation. 

## Figures and Tables

**Table 1:** SILBIONE RTV 4439 qualities (Reprinted from an open-access source) [138].

**Color**	Translucent
**Hardness**	40 Shire
**Elongation**	400%
**Tear Strength**	2.3 N/mm
**Viscosity (at 23 °C)**	8000 mPa∙s
**Polymerization Time**	20 min (at 23 °C)

## Data Availability

Not applicable.
